# Primary Thyroid-Like Follicular Renal Cell Carcinoma: An Emerging Entity

**DOI:** 10.1155/2013/687427

**Published:** 2013-02-21

**Authors:** S. Malde, I. Sheikh, I. Woodman, D. Fish, P. Bilagi, M. K. M. Sheriff

**Affiliations:** ^1^Department of Urology, Medway Maritime Hospital, Gillingham, Kent ME7 5NY, UK; ^2^Department of Pathology, Maidstone Hospital, Maidstone, Kent ME16 9QQ, UK; ^3^Department of Radiology, Medway Maritime Hospital, Gillingham, Kent ME7 5NY, UK

## Abstract

Primary thyroid-like follicular carcinoma of the kidney is a rare but newly emerging histological variant of renal cell carcinoma RCC, with only nine cases reported in the literature to date. We present a further case of this unique condition, discuss the workup and typical histological findings, and review the literature regarding this rare histological variant.

## 1. Introduction

Renal cell carcinoma (RCC) accounts for 2-3% of all cancers, and its incidence is increasing worldwide [1]. It is the commonest solid renal malignancy, accounting for approximately 90% of all renal cancers, and comprises a number of distinct pathological entities. The World Health Organisation Classification of Tumours [2] introduced several new entities in 2004, such as RCC associated with neuroblastoma and mucinous tubular and spindle cell carcinoma. However, a more recently described histological entity has emerged for which there is currently very little clinical data—thryoid-like follicular carcinoma of the kidney (TLFCK). This rare tumour morphologically resembles follicular carcinoma of the thyroid gland, with follicular structures containing abundant colloid-like material, and currently only nine cases have been reported in the world literature [3–6]. We describe a further case of this rare histological entity and provide a review of the literature.

## 2. Case Report

**Figure 1:**
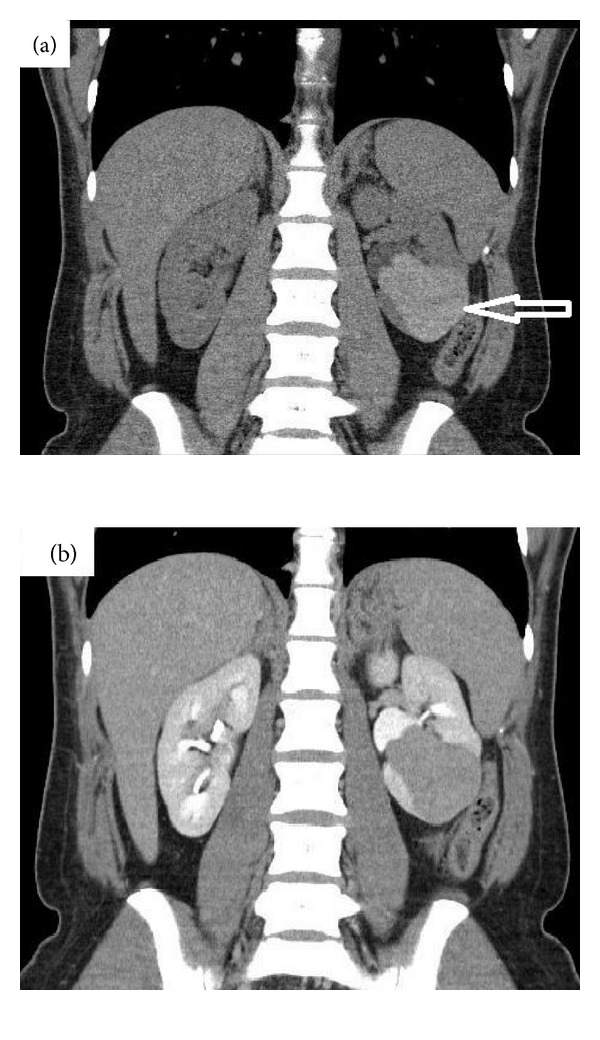
(a) Noncontrast coronal CT scan showing 5.7 × 4.9 × 5.8 cm mass in lower pole of left kidney (see arrow). (b) Postcontrast coronal CT scan showing no measurable enhancement.

A 29-year-old woman presented to her General Practitioner with a long history of left-sided abdominal pain. She had no haematuria, urinary tract infections, or lower urinary tract symptoms, and apart from one previous normal vaginal delivery, no relevant past medical or family history of note. Physical examination of the thyroid and abdomen was normal. Laboratory data, including thyroid-function tests, were within normal limits, and an ultrasound scan revealed a complex multiseptated, and partly cystic, mass arising from the lower pole of the left kidney. Subsequent investigation with a computed tomography (CT) scan confirmed a 5.7 × 4.9 × 5.8 cm lobulated, low-attenuation lesion in the lower pole of the left kidney, with no measurable enhancement following contrast administration (see Figure 1). There was no evidence of renal vein involvement, lymphadenopathy, or metastatic disease.

**Figure 2:**
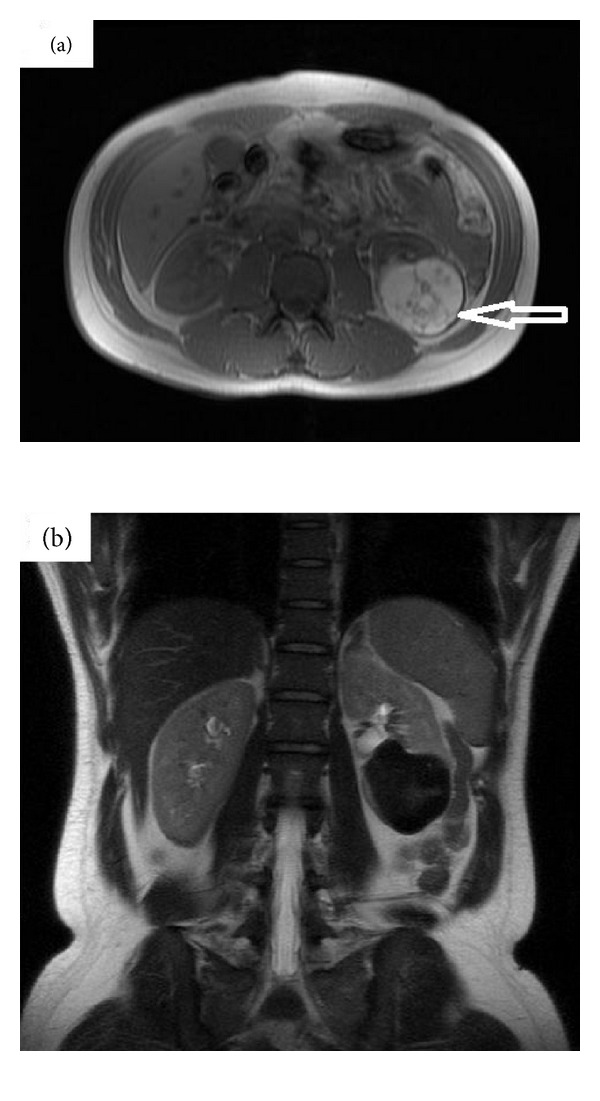
(a) T1-weighted MRI in axial section showing left renal mass with bright signal (see arrow). (b) Left renal lesion is black on this T2-weighted coronal section MRI. No haemorrhagic component was seen.

A magnetic resonance imaging (MRI) scan was performed to determine whether there was a haemorrhagic component, but this was not seen. There was no enhancement following gadolinium injection (see Figure 2). 

In view of the uncertainty regarding the cause of this renal mass, it was decided to proceed with renal biopsy prior to undertaking radical nephrectomy. This showed epithelial tissue with a follicular structure with follicles containing eosinophilic material, and in view of the rare possibility of a primary epithelial renal tumour, nephrectomy was advised. 

She subsequently underwent a left laparoscopic radical nephrectomy without complication. 

## 3. Materials and Methods

The specimen was fixed in 10% buffered formalin and embedded in paraffin wax. Sections 3 *μ*m thick were cut, although this was performed with some difficulty due to the high colloid content. Immunohistochemistry was carried out using standard immunohistochemical techniques.  Table 1 shows the immunohistochemistry panel that was performed. 

## 4. Results 

**Figure 3:**
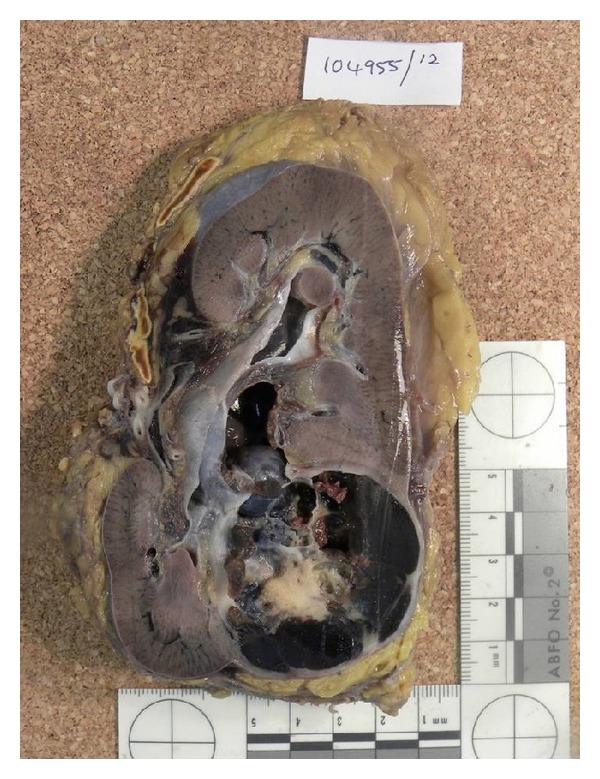
Left kidney demonstrating a 6.5 cm × 4.5 cm well-defined, multiloculated cystic mass within the lower renal pole. The cut surface of the tumour is brown with a central, solid white area.

Macroscopically, the radical nephrectomy specimen demonstrated a single 6.5 cm × 4.5 cm multiloculated cystic mass within the lower renal pole. The cut surface revealed brown gelatinous material with a central solid white area. Ureteric, renal vein and perinephric fat invasion was absent. The background parenchyma was unremarkable (see Figure 3).

**Figure 4:**
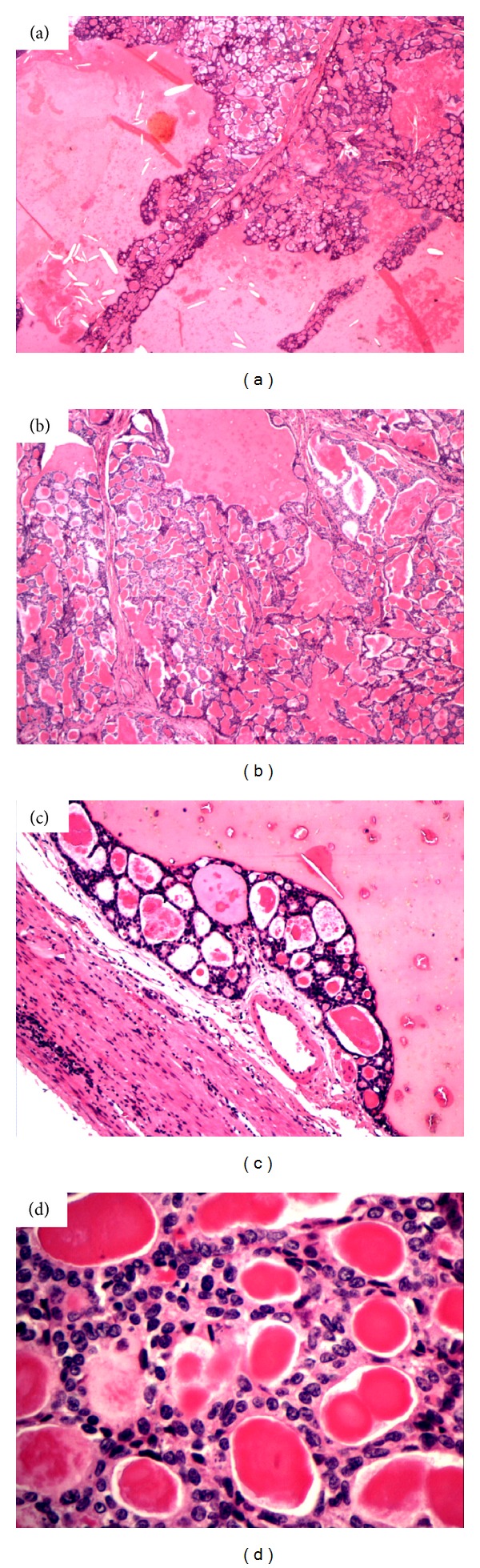
Histological appearance of the tumour. (a) ×2 Follicular areas with fibrous septa and lakes demonstrating clefting. (b) ×4 Fibrous septa and variably sized follicles. (c) ×10 Compressed rim of parenchyma with follicles showing variable appearance of colloid-like material at the edge of a colloid-like lake. (d) ×40 Nuclei demonstrating a fine chromatin pattern with inconspicuous nucleoli.

Microscopically, the tumour was composed of macro- and microfollicles of varying sizes associated with abundant eosinophilic colloid-like material. The follicles were lined by follicular cells with finely granular cytoplasm and round to ovoid nuclei showing fine chromatin pattern and inconspicuous nucleoli (Fuhrman nuclear grade 1). Slender fibrous septae associated with lakes of colloid-like material with cleft artefact were also present. There was little pleomorphism and mitoses were scarce. Coagulative necrosis, calcification, and lymphoid aggregates were absent, and no features of papillary carcinoma of the thyroid were seen (See Figure 4). 

Immunohistochemically, the tumour cells showed positive staining for epithelial membrane antigen (EMA), vimentin, CD10 (focally), and CK7 (focally). Importantly, staining for thyroid transcription factor 1 (TTF-1), thyroglobulin (Tg), CD117, and CK20, was negative confirming that this was not a metastatic thyroid carcinoma but a primary thyroid-like follicular renal cell carcinoma.

The final TNM stage with combined imaging was T1bN0M0.

## 5. Discussion

Primary thyroid-like follicular carcinoma of the kidney (TLFCK) is a rare entity, with only nine cases reported in the literature to date (see Table 2) [3–6]. First described in 2004 [7], several cases have emerged since. Although the majority of cases were low grade with an indolent course, one presented with renal hilar lymph node involvement (tumour diameter 3.5 cm) [4] and another had widespread retroperitoneal lymph node and lung metastases at presentation (tumour diameter 6.2 cm) [5], highlighting the low but distinct malignant potential of these tumours. Despite this, all nine patients are alive and disease-free at a mean follow-up of 28 months. 

The unique histological features of this tumour are identical to the findings in the present report, with widespread microfollicles and macrofollicles containing abundant colloid-like material, bearing a striking resemblance to follicular carcinoma of the thyroid gland. 

Thyroidisation of the kidney has been described in patients with chronic pyelonephritis, obstructive uropathy, or end-stage renal disease, in which renal tissue demonstrates a thyroid-like appearance characterised by atrophic distal tubules and colloid-like hyaline casts [8, 9]. However, this is a benign phenomenon that is typically bilateral and widespread, as opposed to TLFCK which is well circumscribed and occurs in patients without renal disease, as in our case. 

It is possible to have similar histological findings of colloid-like material and follicular structures in the more well-known subtypes of renal cell carcinoma, such as clear cell carcinoma, oncocytoma, tubules of papillary renal cell carcinoma, and metanephric adenoma [4]. However, this is rare, and if present, it is usually focal and is low volume in nature. In contrast, TLFCK is composed primarily of follicles and abundant colloid-like material with none of the other histological features of these more common RCC subtypes. 

The differential diagnosis of this unusual histological finding includes metastatic follicular carcinoma from a thyroid primary, metastases from an ovarian teratoma composed of thyroid tissue (struma ovarii), or a rare primary thyroid-like follicular carcinoma arising in the kidney. These will be discussed in turn. 

Carcinomas of the thyroid gland, both follicular and papillary, very rarely metastasise to the kidney. Only sixteen cases of metastatic follicular thyroid carcinoma to the kidney have been reported, all but one occurring in the presence of widespread metastatic disease involving other organs [10–12]. In each case, there was clinical or radiological confirmation of a primary thyroid malignancy, and all these tumours demonstrated positive immunoreactivity to TTF-1 and Tg. In contrast, negative immunoreactivity to TTF-1 and Tg, in addition to negative clinical thyroid investigations and the absence of metastatic disease elsewhere, confidently excludes the possibility of a metastatic thyroid follicular carcinoma in our case. 

Struma ovarii, an ovarian teratoma composed mainly of thyroid tissue, accounts for 2% of all ovarian tumours [13]. They are rarely malignant and only metastasize in 5% of cases, commonly to the peritoneum or liver [14]. To our knowledge, there are no cases of struma ovarii metastasising to the kidney. In any case, pelvic imaging would be expected to reveal an ovarian tumour, and the tissue would have positive immunoreactivity to TTF-1 and Tg [15]. Both of these features were absent in our case, and combined with the fact that our patient had an uneventful pregnancy and childbirth 18 months previously, this is an extremely unlikely possibility. The unique histopathological features of the present case are consistent with previous reports of a rare primary thyroid-like follicular renal cell carcinoma. 

In conclusion, we report a rare histological subtype of RCC with unique morphologic and immunohistochemical features. Based on the previously reported cases, these tumours generally have an indolent course but there is a distinct malignant risk, and so complete excision remains the treatment of choice. It is important to exclude metastatic disease from a thyroid primary both immunohistochemically and clinically, as this will have an impact on prognosis and treatment. 

## Figures and Tables

**Table 1:** Immunohistochemical profile of the renal tumour.

Antibody	Result
EMA	Positive
Vimentin	Positive
CD10	Focally
CK7	Focally
TTF-1	Negative
Thyroglobulin	Negative
CD117	Negative
CK20	Negative

**Table 2:** Characteristics of the currently reported cases of thyroid-like follicular carcinoma of the kidney.

Cases	Age (years)/sex	Presentation	Tumour size (cm)	TNM stage	Disease-free at follow-up (months)
Soo et al. [3]	32/F	Incidental	11.8	pT2NX	6
Amin et al. [4] case 1	53/F	Incidental	2.1	pT1aNX	54
Amin et al. [4] case 2	29/F	Incidental	1.9	pT1aNX	84
Amin et al. [4] case 3	45/M	Incidental	3.5	pT1aN1	17 (then lost to follow-up)
Amin et al. [4] case 4	83/M	Incidental	2.1	pT1aNX	48
Amin et al. [4] case 5	35/M	Incidental	3.0	pT1aNX	20
Amin et al. [4] case 6	50/M	Incidental	4.0	pT1aN0	7
Dhillon et al. [5]	34/F	Visible haematuria and flank pain	6.2	pT1bN2M1	3
Khoja et al. [6]	31/F	Visible haematuria and flank pain	4	pT1aN0	21
Present case	29/F	Abdominal pain	6.5	PT1bN0M0	4
